# Biocompatibility and Electrical Stimulation of Skeletal and Smooth Muscle Cells Cultured on Piezoelectric Nanogenerators

**DOI:** 10.3390/ijms23010432

**Published:** 2021-12-31

**Authors:** Andreu Blanquer, Oriol Careta, Laura Anido-Varela, Aida Aranda, Elena Ibáñez, Jaume Esteve, Carme Nogués, Gonzalo Murillo

**Affiliations:** 1 Departament de Biologia Cel.lular, Fisiologia i Immunologia, Facultat de Biociencies, Universitat Autonoma de Barcelona, 08193 Bellaterra, Barcelona, Spain; Andreu.BlanquerJerez@fgu.cas.cz (A.B.); oriol.careta@uab.cat (O.C.); laura.anidovarela67@gmail.com (L.A.-V.); elena.ibanez@uab.cat (E.I.); 2 Instituto de Microelectrónica de Barcelona, IMB-CNM (CSIC), Til·lers s/n, Campus UAB, 08193 Bellaterra, Barcelona, Spain; aida.aranda@e-campus.uab.cat (A.A.); jaume.esteve@imb-cnm.csic.es (J.E.)

**Keywords:** stimulation, nanogenerators, muscle cells, piezoelectric, ZnO, electroceutical, bioelectronics, nanosheets, biomedical, biocompatibility

## Abstract

Nanogenerators are interesting for biomedical applications, with a great potential for electrical stimulation of excitable cells. Piezoelectric ZnO nanosheets present unique properties for tissue engineering. In this study, nanogenerator arrays based on ZnO nanosheets are fabricated on transparent coverslips to analyse the biocompatibility and the electromechanical interaction with two types of muscle cells, smooth and skeletal. Both cell types adhere, proliferate and differentiate on the ZnO nanogenerators. Interestingly, the amount of Zn ions released over time from the nanogenerators does not interfere with cell viability and does not trigger the associated inflammatory response, which is not triggered by the nanogenerators themselves either. The local electric field generated by the electromechanical nanogenerator–cell interaction stimulates smooth muscle cells by increasing cytosolic calcium ions, whereas no stimulation effect is observed on skeletal muscle cells. The random orientation of the ZnO nanogenerators, avoiding an overall action potential aligned along the muscle fibre, is hypothesised to be the cause of the cell-type dependent response. This demonstrates the need of optimizing the nanogenerator morphology, orientation and distribution according to the potential biomedical use. Thus, this study demonstrates the cell-scale stimulation triggered by biocompatible piezoelectric nanogenerators without using an external source on smooth muscle cells, although it remarks the cell type-dependent response.

## 1. Introduction

The development of smart materials for biomedical applications has become one of the most interesting research fields in recent years. Smart materials can be defined as materials that have one or more properties that can be changed in a controlled fashion by external stimuli, such as stress, temperature, moisture, pH, and electric, magnetic or ultrasonic fields [1,2,3,4,5]. Piezoelectric materials are smart materials that are able to generate a voltage when mechanical stress is applied, and vice-versa. Among all piezoelectric materials known, ZnO has attracted much interest because of its versatility, unique properties and multiple applications, including microelectronics, biosensors and tissue engineering [6,7,8]. The potential use of ZnO in the biomedical field is wide, with a high range of structures, from bulk material to different types of nanoparticles. In fact, it is already used in healthcare and cosmetic applications [8]. ZnO has been classified as a generally recognized safe material by the Food and Drug Administration (FDA) of the United States. However, there is still controversy about the cytotoxic effect of ZnO nanostructures, with different results depending on cell types and nanostructures, which should be elucidated before their use in biomedical applications.

Zinc is an essential micronutrient and a vital cofactor that plays an important role in tissue metabolism. It is indispensable in numerous Zn-dependent proteins involved in transcriptional regulation, DNA repair and extracellular matrix regulation, among others [9]. Controlled low concentrations of Zn are present in the cytosol as free ions that have been identified as secondary messengers capable of interacting with proteins to regulate intracellular pathways. Therefore, the presence of low concentrations of Zn is beneficial to cells, although high concentrations could be harmful [10]. In this regard, the use of Zn-based biodegradable materials is considered a good choice for tissue regeneration.

ZnO has been demonstrated to be a considerably good antibacterial material, which is an important property for very different kinds of biomedical applications. Several authors have investigated this antibacterial effect on *Staphylococcus epidermidis* and *Staphilococcus aureus*. Both types of bacteria could infect the patient during surgery when present in implants and medical devices. The infection and the consequent immunological response could result in implant failure, causing serious problems for the patient. Several authors have demonstrated a decrease in bacterial adhesion and a significant decrease in bacterial viability on ZnO samples [11,12].

Controversial results on mammalian cells’ interaction with ZnO nanostructures and nanoparticles have been reported depending on the specific shape of ZnO structures and the cell type. Hydroxyapatite-ZnO composites with polished surfaces allowed cell attachment, spreading and proliferation of osteoblasts and fibroblasts [13]. Results for ZnO nanorods showed that adhesion and, thus, viability of anchorage-dependent cells was reduced, indicating that these substrates cannot be used for biomedical implants when cell adhesion is needed [14,15]. In contrast, ZnO nanoflowers improved mouse MC3T3-E1 osteoblast adhesion, proliferation and differentiation when compared with ZnO films, although cell adhesion and differentiation were higher on polystyrene control [16]. All these results suggest that ZnO biocompatibility may depend on its structure and, also, the specific cell type. In this regard, it is necessary to analyse each nanostructure with the cell type of choice, according to the potential application, before considering its use as an implant or device candidate.

Nowadays, nanostructured ZnO is very popular for its application as piezoelectric nanogenerators (NGs). Millimetre-scale NGs have already been used for biological applications, including sensors and generators. However, NGs have also a great potential for electrical stimulation, at the cell scale, of excitable cells like osteoblasts, neurons and muscle cells [17]. In a previous paper, we demonstrated the cytocompatibility of ZnO nanosheets (NSs) on osteoblasts and macrophages, and their use as NGs to induce a cellular response to the local electric fields generated by the piezoelectric NSs. The inherent forces produced by the cells were able to induce a piezopotential without the use of external stimulation [18]. Electric stimulation of different cell types has an important effect on cell regeneration and metabolism. Muscle cells respond to electric fields and induce the contraction of muscle tissue. Thus, the local electric field generated by ZnO NSs would potentially improve the regeneration and rehabilitation of muscle tissue, with several future applications in chronic illnesses such as muscle atrophy, wasting and aging [19,20].

In this study, we analyse the potential application of ZnO NSs on two muscle cell lines, one derived from mouse skeletal muscle and the other from rat smooth muscle. Both cell types are electrically excitable cells. We studied the cytocompatibility of ZnO NSs and the NGs–cells interaction by analysing cell viability, morphology, cytoskeleton distribution and proliferation over time. In addition, we studied the effect of electrical fields generated by the NGs on cell response by measuring intracellular calcium changes.

## 2. Results

### 2.1. NG Characterization and Zn Ions Release over Time

**Figure 1:**
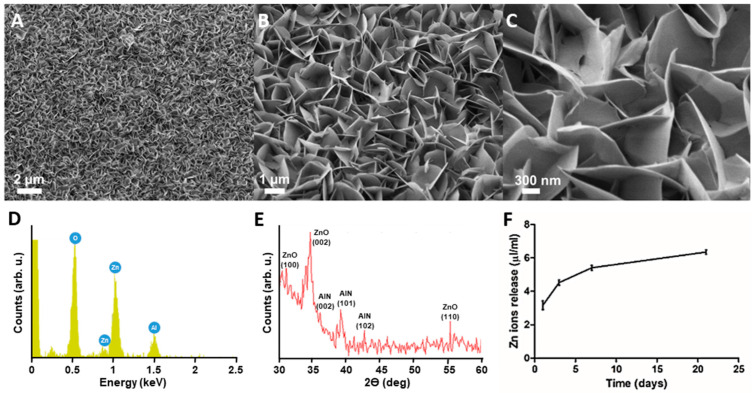
Characterization of ZnO NG arrays. SEM images of NSs grown for 9 h at 80 °C over an AlN layer of 100 nm at different amplification magnitudes: 1 KX, 5 KX and 20 KX (**A**–**C**); XRD measurement showing the high crystallinity of the NSs and the preferential orientation along (002) (**D**); Measurement EDS at the surface of the NSs, showing the presence of Zn, O and Al (**E**). Concentration of Zn ions released from the ZnO NSs array over time in culture medium (**F**).

The grown ZnO NSs, which form the NG array on the AlN-coated coverslip, showed an optimized aspect ratio to be used for cell stimulation, with a mean thickness of 23 ± 7 nm (Figure 1A–C and Figure S1). Due to the reduced thickness of the AlN layer and the ZnO NSs, the resulting glass coverslips, once covered with the NG array, are translucent, facilitating the optical microscope inspection. In addition, the wurtzite crystal structure and preferential growth along (002) orientation were validated with X-ray diffraction (XRD) analysis (Figure 1D). An energy-dispersive spectroscopy (EDS) analysis confirmed the presence of Zn and O on the NS composition before immersion in cell culture medium (Figure 1E). The amount of Zn ions released from the NSs after immersion in the cell culture medium is shown in Figure 1F. Zn ions quantification using inductively coupled plasma mass spectrometry (ICP-MS) indicated a time-dependent dissolution, with a progressive increase over time. The quickest release was observed after 3 days in culture, reaching a concentration of 4.5 µg mL^−1^ of Zn in a volume of 1 mL. Zn concentration in the culture medium increased to 5.4 µg mL^−1^ after 7 days, reaching 6.3 µg mL^−1^ after 21 days.

### 2.2. ZnO Cytocompatibility for Smooth Muscle Cells

**Figure 2:**
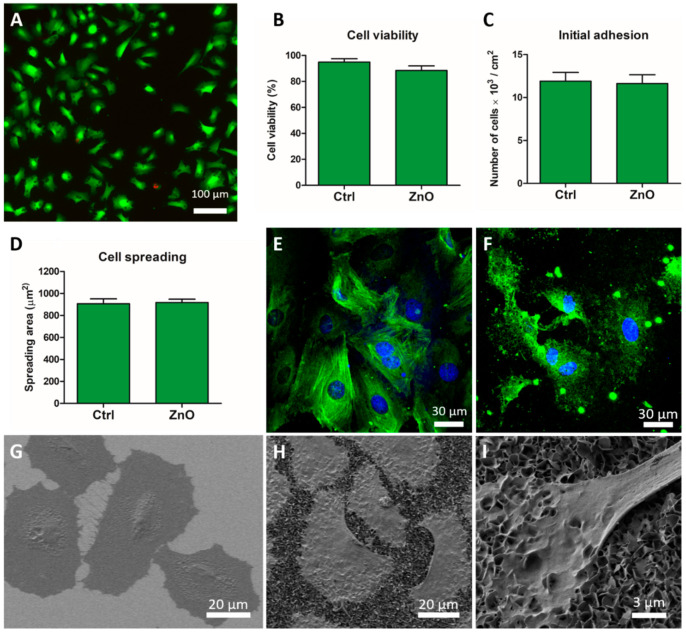
Smooth muscle (A7r5) cells grown on ZnO NG arrays. (**A**) Live (green) and dead (red) A7r5 cells cultured on ZnO NG array. (**B**) Percentage of viable A7r5 cells after 24 h in culture on ZnO NG arrays and glass coverslip (Ctrl), evaluated using Live/Dead viability/cytotoxicity kit. (**C**) Number of adhered cells and (**D**) mean spreading area of cells after 24 h in culture on ZnO NG arrays and glass coverslip (Ctrl). Confocal images of α-smooth muscle actin immunodetection (green) and nuclei (blue) of cells growing on glass coverslip (**E**) and ZnO NG arrays (**F**) for 24 h. SEM images of well-spread A7r5 cells on glass coverslip (**G**) and ZnO NG arrays (**H**,**I**) for 24 h. 2.3. ZnO cytocompatibility for skeletal myoblasts.

Cytocompatibility of ZnO NG arrays for smooth muscle (A7r5) cells was analysed in terms of several biological parameters such as cell viability, adhesion, morphology and proliferation. Cell viability was analysed by quantifying the number of live cells using the Live/Dead kit, presenting live cells with esterase activity in green and dead cells in red (Figure 2A). The percentage of live cells was higher than 88%, without significant differences when compared with the same cells grown on glass coverslips (control sample) (Figure 2B). In addition, no significant differences were found in the initial number of cells adhered and the mean spreading area of the cells after 24 h in culture between A7r5 cells growing on ZnO NG arrays and glass coverslips (Figure 2C,D).

A7r5 cells’ interaction with ZnO NG arrays and the morphology of A7r5 growing on ZnO NG array were analysed after 24 h in culture using confocal laser scanning microscope (CLSM) and scanning electron microscope (SEM). Immunofluorescence analysis of α-smooth muscle actin showed different distribution of actin fibres in A7r5 cells grown on ZnO NG arrays and glass coverslip. As shown in Figure 2E,F, cells grown on coverslip presented well-defined actin fibres crossing the cell from end to end, whereas cells grown on a ZnO NG array presented shorter actin bundles adapted to the topography of the NSs. However, cell spreading indicated that cells were well adhered to ZnO NSs without disturbing cell growth, allowing a close interaction between cells and NGs. A similar well-spread morphology was observed for cells growing on the ZnO NG arrays using SEM. Cell morphology analysis by SEM indicated that cells grown on a ZnO NG array and glass coverslip displayed a polygonal shape and a flattened morphology (Figure 2G,H), in close contact with the NGs (Figure 2I).

The effect of ZnO NGs on skeletal muscle (C2C12) cells was analysed in terms of cell viability, adhesion, morphology and proliferation, as for the smooth muscle cells. In addition, we analysed myotubes formation by C2C12 cells fusion on the ZnO NG array.

**Figure 3:**
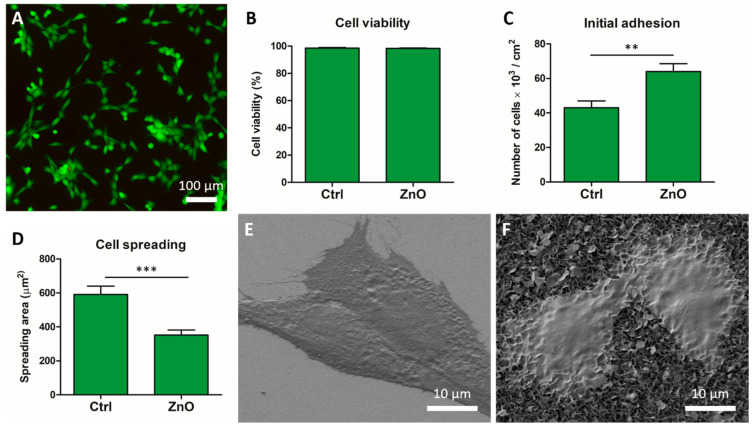
Skeletal myoblasts (C2C12) cells grown on ZnO NG arrays. (**A**) Live (green) and dead (red) C2C12 cells cultured on ZnO NG array. (**B**) Percentage of live cells after 24 h growing on ZnO NG arrays and glass coverslips (Ctrl), evaluated using Live/Dead viability/cytotoxicity kit. (**C**) Number of adhered cells and (**D**) mean spreading area of cells after 24 h in culture on ZnO NG arrays and glass coverslip (Ctrl). Statistically significant differences are marked by two asterisks (*p* < 0.01) or three asterisks (*p* < 0.001). SEM images of C2C12 cells morphology on glass coverslip (**E**) and ZnO NG arrays (**F**) after 24 h in culture.

Quantification of live cells using the Live/Dead kit showed that the viability of C2C12 cells growing on ZnO NG arrays was higher than 98% (Figure 3A), and no significant differences were observed between ZnO samples and glass coverslip (Figure 3B). Statistically significant differences were found in the initial number of cells adhered after 24 h, being higher on the ZnO NG arrays (64 ± 4 × 10^3^ cells/cm^2^) compared to glass coverslip (43 ± 4 × 10^3^ cells/cm^2^) (Figure 3C). However, the mean spreading area of cells was significantly reduced for cells growing on ZnO NG arrays (351 ± 30 µm^2^), when compared to cells growing on glass coverslip (591 ± 49 µm^2^) (Figure 3D).

### 2.3. Cell Morphology

**Figure 4:**
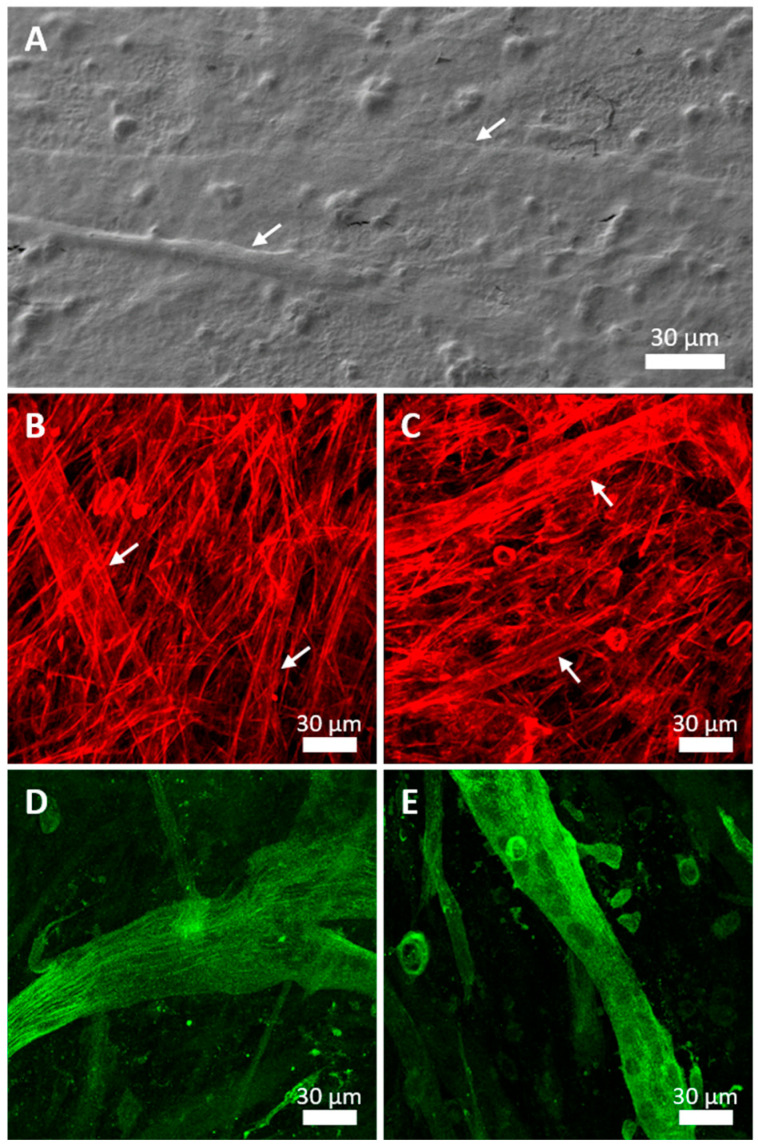
SEM images of differentiated C2C12 cell after 7 days growing on ZnO NG arrays (**A**). CLSM images of skeletal myoblasts (C2C12) cells differentiated after 7 days in culture on glass coverslip (**B**,**D**) and ZnO NG arrays (**C**,**E**). Stress fibres immunodetection is shown in red (**B**,**C**) and α-actinin immunodetection is shown in green (**D**,**E**). Skeletal muscle myotubes (arrows) were formed after 7 days in culture on both samples.

Cell morphology was evaluated using SEM after 24 h and 7 days in culture. After 24 h, individualized C2C12 cells showed a well-spread morphology on both ZnO NG array and glass coverslip, although cells growing on control glass seemed to be wider, as expected for the spreading area measurements (Figure 3E,F). Cytoplasmic extensions were visible, with a close interaction with ZnO NG arrays. After 7 days, a monolayer of cells was observed covering the whole ZnO NG array and differentiated myotubes were also observed (Figure 4A).

Stress fibres distribution after 7 days in culture was analysed using CLSM. C2C12 cells showed clear stress fibres, without evident differences between ZnO NG arrays and coverslip samples (Figure 4B,C).

After 7 days in culture with the differentiation medium, it was possible to observe the fusion of several cells forming myotubes on both samples. To confirm that the structures observed were myotubes, the presence of sarcomeric α-actinin, a component of Z-disk in differentiated skeletal muscle myotubes, was analysed. Images showed that α-actinin were evident in differentiated myotubes in both substrates, without clear differences between them. Longitudinal myofilament bundles crossing the whole cell were detected (Figure 4D,E). In addition, a monolayer of undifferentiated myoblasts without α-actinin labelling or diffused signal were visible.

### 2.4. Immunological Response

**Figure 5:**
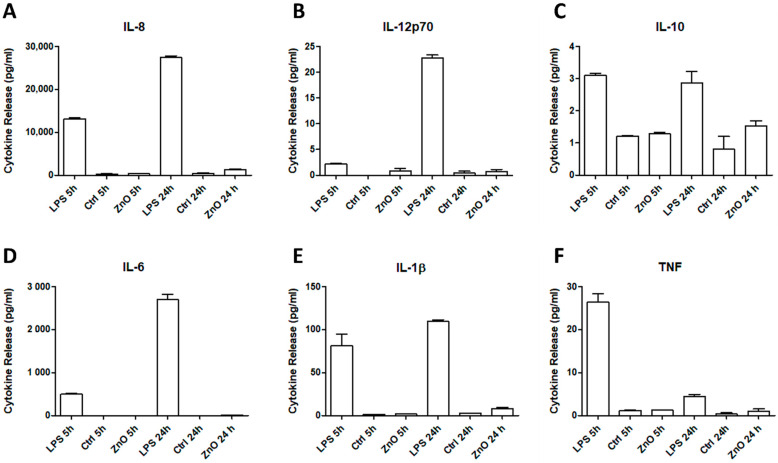
Cytokine release by macrophages analysed by the CBA test after 5 and 24 h of culture. Six types of cytokines involved in inflammation, IL-8 (**A**), IL-12p70 (**B**), IL-10 (**C**), IL-6 (**D**), IL-1β (**E**) and TNF (**F**), are shown in the presence of lipopolysaccharide (LPS, positive control), ZnO NG array, and a negative control.

To find out whether the ZnO NG arrays activate the secretion of inflammatory cytokines, we analysed the presence of six cytokines involved in inflammation using the cytometric bead array (CBA) test. Although all six cytokines quantified are important in the inflammatory response, each one plays a different role and the secretion by macrophages could be different. In general, IL-8, IL-12p70, IL-6, IL-1β and TNF are considered pro-inflammatory cytokines, whereas IL-10 is considered an anti-inflammatory cytokine. Quantification results showed that no significant increase in cytokines concentration was observed after 5 h and 24 h of macrophages culture on the ZnO NG array (Figure 5). On the contrary, the presence of lipopolysaccharide (LPS) in the cell culture medium stimulated the secretion of all cytokines tested.

### 2.5. Intracellular Calcium Changes

**Figure 6:**
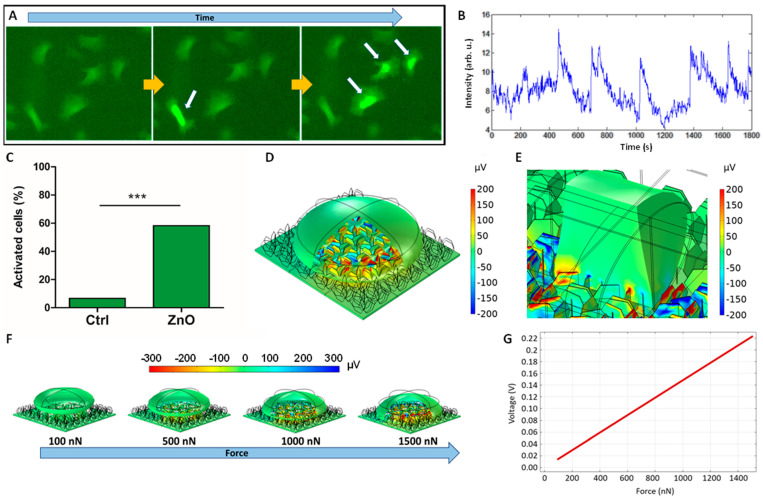
Frames of time-lapse video showing florescence due to calcium influxes in smooth muscle (A7r5) cells (**A**). Intensity of fluorescence indicating calcium influx in a certain A7r5 cell at timepoints: 5 min, 6 min and 7 min (**B**). Percentage of activated smooth muscle (A7r5) cells which undergo changes in intracellular calcium concentration (**C**); statistically significant differences are marked by three asterisks (*p* < 0.001). COMSOL simulations with the piezopotentials generated by a smooth muscle cell (**D**) and cylinder-like cell (**E**), corresponding to a sarcomere of a skeletal muscle fibre, when exerting a force of 1 µN. Simulation of smooth muscle cell shape and electric potential distribution (**F**) and values of the potential at the membrane (**G**) for different muscle forces.

Calcium is a second messenger involved in several intracellular pathways, such as muscle contraction. Skeletal muscle cells and smooth muscle cells exposed to electrical stimulation undergo changes in intracellular calcium levels (Figure 6A,B), which induces muscle contraction.

Results in smooth muscle cells after 24 h in culture showed that A7r5 cells grown on the ZnO NG array presented 58% of cells with calcium transients, whereas only 7% of cells presented calcium transients on glass coverslip (Figure 6C). By contrast, no significant differences in intracellular calcium changes were detected between C2C12 cells grown for 7 days on ZnO NG arrays and glass coverslip (Video S1 in Supporting Information).

### 2.6. Finite Element Modelling

Finite element modelling (FEM) has been used to estimate the piezopotential that the NG array produces when cells adhere to the top of the material (Figure 6D). The range of forces which cells exert when they attach and move goes from 0.1 nN to 100 nN [21,22,23]. In case of muscle cells, the forces produced can be in the µN range [24]. As a representative value, a force of 1 µN perpendicular to the top of the cell surface has been applied for a smooth muscle cell already adhered (flattened shape, e.g., day 7 of culture). A variety of values of piezopotentials for each NSs positioned not perpendicular to the membrane can be observed, being the maximum voltages around +200 µV and −200 µV in the cell membrane. Considering the absolute value of the piezopotential generated for each NG, there are no important differences in magnitude. This voltage depends on the cell force, and the NS size. The size of the NSs has been optimized for cell stimulation by inherent cell forces, obtaining the best result for 9 h of growth at 80 °C using an AlN seed layer of 100 nm (unpublished work). In addition, a model of a myotube with a stretching force of 1 µN, perpendicular to the cross sections of the cylinder, has been simulated. Figure 6E shows the voltage in a specific region that represents a sarcomere as unit of contraction of a skeletal muscle fibre. As can be shown, the force in that direction generates similar voltages in the cell membrane, ranging from +200 µV to −200 µV, although higher voltages can be obtained along the NSs. However, due to the overall charge cancelation, the transversal potential difference between both cross-sectional faces is lower than 1.13 µV. This potential is inferior to the local potentials found for the smooth cells, where the overall direction of the potential difference seems not to be critical for the cell activation. Figure 6F,G shows the simulations of a smooth muscle cell with the electric potential distribution and the values of the electric potential at the membrane for different muscle forces. The electric potential values go from around −300 mV to 300 mV at the cell membrane, for normal cell forces in the range of the 100 nN to 1500 nN.

## 3. Discussion

ZnO nanostructures have attracted remarkable attention as smart piezoelectric materials for biomedical applications. However, several authors have reported controversial results on the biocompatibility of different ZnO nanostructures and nanoparticles with different cell types. In this regard, the morphology of ZnO plays an important role for cytotoxicity. In the present work, ZnO NG arrays based on ZnO NSs were fabricated for biomedical purposes. The hydrothermal method to grow NSs was optimized to be performed on glass coverslips and to obtain a thin layer of NSs with the optimal NS thickness to ensure the piezoelectric effect. All the samples obtained presented the same area and were translucent enough to visualize the cells under an optical microscope.

On the other hand, biodegradability is another important property of ZnO. New biodegradable devices have a great interest in tissue engineering, although degradable products should not cause undesirable effects. Su et al. (2018) reported that cytotoxicity of ZnO nanoparticles is mainly due to increased intracellular Zn ions as a result of ZnO solubility [10]. For this reason, we decided to quantify the concentration of Zn ions released from the ZnO NSs array over time. The results obtained showed that concentrations reached 5.4 µg mL^−1^ and 6.2 µg mL^−1^ after 7 and 21 days, respectively. It has been stated that concentrations lower than 27 µg mL^−1^ of Zn ions allowed human neuroblastoma cells proliferation [25]. Specific results for smooth muscle cells demonstrated that less than 5.3 µg mL^−1^ of Zn ions have beneficial effects on human aorta smooth muscle cell adhesion, spreading, viability, proliferation and migration [26]. However, different smooth muscle cells from different species respond differently, showing inhibited proliferation of smooth muscle cells from carotid artery of Wistar rats at 2.5 µg mL^−1^ of Zn ions or significantly reduction of human prostatic smooth muscle cells proliferation above 16.7 µg mL^−1^ [27]. Taking into account these previous results, we expected that the concentration of Zn ions release from ZnO NSs array would not reduce the viability of smooth muscle cells and skeletal muscle cells. To elucidate this point, we analysed the viability, adhesion, proliferation and differentiation of the two muscle cell types on ZnO NG arrays up to 7 days in culture. In both cell types, cells presented high viability and were able to proliferate and differentiate on ZnO NG arrays. These results are in agreement with our previous results when analysing cytotoxicity of ZnO NG arrays for Saos-2 cells and macrophages [18]. Moreover, the Zn ions released from the ZnO NG arrays did not have any negative effect either on smooth muscle cells or on skeletal muscle cells.

To go further in the analysis of biocompatibility, we analysed the immunogenic response to ZnO NG arrays. The biological response to corrosion products from the devices can be responsible for inflammatory reactions and can induce the formation of a non-adherent fibrous capsule surrounding the device, which could end in chronic inflammation [28]. We analysed the release of six cytokines involved in inflammation by macrophages when cultured in the presence of ZnO NG arrays and controls. Results indicated that after an incubation of 5 and 24 h with ZnO NG arrays, no significant amounts of inflammatory cytokines were secreted compared with the negative control. However, the secretion of cytokines was high when macrophages were incubated with LPS as positive control. It has been reported that the inflammatory response activation depends on the particle and ion concentration. In this regard, we can correlate the ions’ release after 24 h with the inflammatory response and assume that a concentration of 3.1 µg mL^−1^ of Zn ions does not activate it.

Surface topography can modulate cell adhesion, morphology and the interaction between cells and material. In addition, topography and stiffness could influence the differentiation of cells on biomaterials and devices. The results showed that both types of muscle cells adhered well to ZnO NSs, with the cytoskeleton adapted to the topography and cytoplasmic prolongations connected to NSs. The close interaction between cells and the NGs is necessary for the induction of a piezoelectric effect. In the case of C2C12 cells, we found an increase in the number of cells initially adhered to ZnO NG arrays compared to glass coverslips, although the spreading area of the adhered cells was lower in ZnO NG arrays. The C2C12 cells are able to proliferate as soon as they adhere to a surface and the population doubling time is very short. Therefore, due to the proliferative rate of C2C12 cells, no differences were observed after 7 days in culture. Besides the proliferation, skeletal muscle cells need to differentiate to create functional myotubes. After 4 days, myoblasts fuse in response to the differentiation medium, producing syncytia with several nuclei [29]. Ciofani et al. (2012) analysed the differentiation of H9c2 cells on ZnO nanowires as a model for skeletal muscle cells, and observed that cells displayed a disordered arrangement, without showing the typical tubular shape of H9c2 myotubes [30]. They suggested that the stiffness or the nanotopography of ZnO nanowires was cause for the prevention of myotubes formation. By contrast, our results showed that C2C12 cells were able to differentiate and form myotubes on ZnO NGs, similar to those formed when grown on control samples. The formation of myotubes is a necessary step for further functionality of muscle cells, including myotubes contraction.

Both smooth muscle cells and skeletal muscle cells are able to respond to electric stimulation by voltage-gated calcium channels (VGCC). Plasma membrane depolarization caused by the electric fields opens the VGCC and, subsequently, increases the intracellular calcium concentration. In our study, we analysed the intracellular calcium changes in cells grown on the ZnO NG arrays and found different results depending on the cell type tested. Intracellular calcium transients were detected in smooth muscle cells, showing a significant increase on ZnO NG arrays (i.e., 58% of activated cells compared to 7% in the control), whereas no differences were detected in skeletal muscle cells. Results suggest that piezoelectric ZnO NG arrays are able to generate a local electric field that activates the smooth muscle cells. These results are in agreement with our previous results on osteoblasts, where we hypothesized that the mechanical stress produced by cell adhesion is responsible for their own electromechanical stimulation [18]. Similar results were also observed in piezoelectric PVDF nanofiber scaffolds [31]. In addition, FEM simulations also validate the piezoelectric stimulation of smooth muscle cells, that is, the NGs can create local piezopotential distributions, allowing a local activation of the VGCC and the corresponding calcium influx. The simulated voltages generated by the NGs range from around −300 mV to 300 mV at the cell membrane, which are values theoretically high enough to activate the VGCCs at the cell membrane.

By contrast, skeletal muscle cells did not show any effect of the ZnO NG arrays regarding intracellular calcium changes. These results were unexpected, but several factors may be responsible for the lack of cell stimulation. In the literature, several authors have studied the effect of electrical stimulation on C2C12 cells by analysing a wide range of electric fields, frequency and timing of the stimulation. All of them agree that electrical pulses with certain periodicity are necessary to mimic the physiological stimulation in vitro, and that they allow the creation of functional skeletal muscle tissues [20,32]. Indeed, single myoblasts respond to electromagnetic fields by modulation of the intracellular calcium [33]. The effect of electromagnetic fields to myoblasts could be responsible for the creation of functional myotubes. On the other hand, multinucleated myotubes in physiological tissues present a highly unidirectional orientation, which is necessary to produce contracting forces. In vitro, myotube formation loses the natural organization and the myotubes appear randomly distributed. It seems that the lack of cell alignment may alter the functionality of skeletal muscle [34]. In our study, we hypothesized that adherent cell forces would be able to mechanically stress the NGs and induce a local electric field to stimulate muscle cells. Therefore, no external energy sources were used and the potential electric stimulation was generated randomly by the different ZnO NGs of the array. In addition, a single myotube could be stimulated by several NGs at the same time and at different regions of the plasma membrane, depending on the area of the cell pressing on the ZnO NG array during adhesion or movement. This random stimulation in different directions depending on the orientation of each NS could alter the contraction because of the lack of unidirectional electrical stimulation. The randomly generated electric fields by ZnO NGs was validated with the COMSOL simulations performed with a random NG array. As has been reported, the electric field direction is crucial for correct muscle contraction when an electric field is applied [35,36]. The myotube contraction is controlled by the direction of the electric field due to the highly anisotropic electrical properties of skeletal muscle. Moreover, electrical parameters such as frequency, pulse width and voltage amplitude should be controlled to stimulate the skeletal muscle contraction without electrochemical damage or toxicity. Therefore, to allow for a specific stimulation for this type of cells, we should use an NG array with aligned NSs along the perpendicular direction of the myotubes. Unfortunately, the hydrothermal method used for this work does not allow for this alignment. However, research using a micromachined piezoelectric material, such as PVDF, with aligned patterns at the nanoscale (e.g., using stamp or soft-lithography or reactive ion etching) is currently being conducted, with promising potential. In addition, it is possible to use ultrasound signals to wirelessly actuate the NGs in order to electrically stimulate the cells. The results provided by this work allow the study of a family of groundbreaking developments for a personalized and minimally invasive nanomedicine.

## 4. Materials and Methods

### 4.1. NG Synthesis and Optimization

NG arrays based on ZnO NSs were synthesised using a hydrothermal growth at mild conditions. In order to allow growth on the NS shape, a thin-film layer of 100 nm of AlN was deposited by using radio-frequency (RF) sputtering on top of glass coverslips. An aqueous solution containing hexamethylenetetramine (HMTA) and Zn(NO_3_)_2_ was introduced in an amber wide-mouth jar. The coated glass coverslips were placed in the jar, floating on the solution, with the AlN layer facing down. The jar was sealed and introduced in an oven at 80 °C for 9 h. Then, the coverslips were collected, rinsed in deionized water and ethanol and left to dry at room temperature (RT). The growth parameters (i.e., 80 °C for 9 h) were optimized in order to improve the activation effect.

### 4.2. NG Characterization

The grown ZnO NSs on an AlN-coated coverslip were characterized using an SEM Zeiss Auriga at 3 kV and a secondary electrons detector together with an energy-dispersive X-ray spectroscopy (EDS/EDX) detector. EDX measurements were obtained by applying a voltage of 5 kV. In addition, XRD analysis was obtained on a Bruker D8 Advance diffractometer (CuKα, λ = 1.5418 Å) with a bidimensional general area detector diffraction system (GADDS) detector. For this measurement, a 2θ angle ranging from 30° to 60°, a voltage of 40 KV and a current of 40 mA were used.

### 4.3. COMSOL Simulation

We used the piezoelectric physics of COMSOL to simulate the piezopotential generated by NGs based on hexagonal ZnO NSs. The ZnO NSs were created with a thickness of 30 nm and a height of 1000 nm. A layer of 100 nm of AlN was used in the bottom surface. Mechanically, all the substrate was anchored. A force of 1 µN was applied normally to the cell membrane. A tetrahedral mesh with adaptive size (i.e., coarse for cell, medium for substrate and fine for NSs) was used (Figure S2). The piezoelectric origin axes were carefully chosen for each NS, so that they were aligned along their respective c-axis.

### 4.4. Zinc Ions Released from NGs

In order to analyse the biodegradability of the ZnO NG arrays, the amount of zinc ions released from the NGs was quantified. ZnO NG arrays were sterilized in ethanol for 30 min and incubated with 1 mL of DMEM cell culture medium at standard conditions (37 °C and 5% CO_2_). The culture medium was withdrawn after 24 h and after 3, 7 and 21 days, and the zinc concentration was analysed by ICP-MS on an Agilent ICP-MS 7500ce equipment (Agilent Technologies, Santa Clara, CA, USA).

### 4.5. Cell Lines

Two different muscle cell lines were used to test the cytocompatibility of the ZnO NG arrays and to analyse the piezoelectric effect generated by them: C2C12 mouse skeletal myoblasts and A7r5 rat smooth muscle cells (both from ATCC). C2C12 cells were cultured under standard conditions in Dulbecco’s modified Eagle medium (DMEM; Invitrogen) supplemented with 10% foetal bovine serum (FBS; Thermo Fisher Scientific, Waltham, MA, USA). For myoblast differentiation, the growth medium was replaced by a differentiation medium containing DMEM supplemented with 10% horse serum (Thermo Fisher Scientific) after 24 h of cells seeding. The differentiation medium was replaced each 2 days for 7 days. A7r5 cells were grown in DMEM supplemented with 10% FBS under standard conditions.

THP-1 monocyte cells were used to analyse the immunological response to the ZnO NG array. Monocytes were grown in an RPMI 1640 medium (Gibco) supplemented with 25% FBS in standard conditions. To differentiate monocytes into macrophages, 400,000 THP-1 cells were seeded into 24-well plates and treated with 0.16 µM phorbol-12-myristate-13-acetate (Sigma) for 72 h. Then, the cells were washed and incubated in a fresh medium for 24 h before carrying out the experiments.

### 4.6. Cell Viability Assay

Direct cytotoxicity was evaluated by detecting the activity of intracellular esterases using the Life/Dead Viability/Cytotoxicity kit for mammalian cells (Invitrogen), according to the manufacturer’s protocol. Briefly, ZnO NG arrays were cleaned and sterilized with absolute ethanol for at least 1 h. Once sterilized, samples were transferred into 4-well culture plates and 1 × 10^5^ C2C12 cells or 3 × 10^4^ A7r5 cells were seeded on top of each ZnO NG array and cultured for 24 h. In parallel, the same number of cells were seeded on glass coverslips as control substrates. After 24 h, cells were incubated with the kit and images from different regions of the samples were captured on an inverted epifluorescence microscope (Olympus IX7). Each experiment was performed in triplicate and a minimum of 300 cells were analysed per sample.

### 4.7. Initial Cell Adhesion and Spreading Area of Cells

To quantify the number of cells initially adhered on samples and the mean spreading area per cell, images previously captured for cell viability assay were used. The cell number was quantified using the cell counter plugin of Image J software. The spreading area was measured analysing fluorescent calcein using Image J software. For each experiment, at least 15 regions from 3 replicates were analysed.

### 4.8. Cell Morphology Assay

At the end of the cell viability assay, the same samples were prepared for SEM visualization. Cells grown on samples were rinsed twice in phosphate buffered saline (PBS), fixed in 4% paraformaldehyde (PFA; Sigma) in PBS for 15 min at RT and rinsed twice in PBS. Then, the cells were dehydrated by ethanol at increasing concentrations (50%, 70%, 90% and twice 100%) for 8 min each and dried using hexamethyl disilazane (Electron Microscopy Science) for 15 min. Finally, samples were mounted on special stubs and analysed using SEM Zeiss Auriga.

### 4.9. Immunofluorescence Detection of Smooth Muscle Actin on A7r5 Cells

Cytoskeleton distribution in A7r5 cells was analysed by detecting α-smooth muscle actin (α-SMA), a specific protein of smooth muscle cells. A total of 6 × 10^4^ cells were seeded on ZnO NG arrays and glass coverslip controls. After 7 days in culture, the cells were fixed in 4% PFA in PBS for 15 min at RT, and permeabilised and blocked with 0.5% Triton X-100, 0.2% sodium azide and 3% goat serum in PBS for 30 min at RT. Then, the cells were incubated with mouse anti-α-SMA IgG monoclonal-antibody (1:500; Sigma) overnight at 4 °C. The next day, the cells were incubated with Alexa Fluor 488 goat anti-mouse IgG (1:500; Thermo Fisher Scientific) for 2 h at RT in the dark. Finally, samples were incubated with Hoechst 33258 (5 µg/mL; Sigma) for 15 min at RT. ZnO NG arrays and control glass coverslips with stained cells were mounted using ProLong Antifade (Thermo Fisher Scientific) and evaluated with a CLSM (Olympus).

### 4.10. Immunofluorescence Detection of Sarcomeric α-Actinin on C2C12 Cells

Myoblast differentiation was determined by an immunodetection of sarcomeric α-actinin after 7 days in a differentiation culture medium. A total of 1 × 10^5^ C2C12 cells were seeded on ZnO NG arrays and glass coverslip controls. After 7 days in culture, the cells were fixed in 4% PFA in PBS for 15 min at RT, permeabilised with 0.1% Triton X-100 (Sigma) in PBS for 15 min and blocked for 20 min with 1% bovine serum albumin (BSA; Sigma) in PBS at RT. Samples were then incubated with mouse anti-sarcomeric α-actinin IgG monoclonal-antibody (1:500; Abcam) for 1 h at RT and washed with 1% BSA-PBS. Then, the samples were incubated with Alexa Fluor 488 goat anti-mouse IgG (1:500) for 45 min at RT in the dark. Finally, the samples were incubated with Hoechst 33258 (5 µg/mL) for 15 min at RT. ZnO NG arrays and control glass coverslips with stained cells were mounted using ProLong Antifade and evaluated with a CLSM.

### 4.11. Quantification of Inflammatory Cytokines Secretion

THP-1 cells differentiated into macrophages were used to conduct this analysis. ZnO NG arrays were sterilized and placed over a culture of macrophages and maintained in contact with the cells for 5 and 24 h. After each time-point, supernatants were removed and used to quantify cytokines secretion. Six inflammatory cytokines, IL-8, IL-12p70, IL-10, IL-6, IL-1β and TNF, were evaluated by flow cytometry using a CBA (Becton Dickinson, Franklin Lakes, NJ, USA). Cytokine concentration in the supernatant was analysed according to manufacturer’s protocol. A positive control with 1 µg/mL of LPS (Sigma) and a negative control were performed together with samples analysis.

### 4.12. Intracellular Calcium Quantification

A7r5 cells were cultured on samples for 24 h and C2C12 cells were cultured on samples for 7 days in differentiation medium. Then, the cells were loaded with 2 µM Fluo-4 AM (Life Technologies, Carlsbad, CA, USA) in serum free DMEM for 30 min in the dark at RT. The samples were washed with serum-free DMEM and transferred to MatTek dishes with a fresh medium without phenol red. Images were captured using an Olympus IX71 inverted microscope equipped with epifluorescence and heating plate. A time-lapse every 1 s was recorded for 15 min for each sample (Video S1 in Supporting Information). Changes in fluorescent intensity during the time of monitoring were processed and quantified using Image J software (NIH).

### 4.13. Statistical Analysis

The quantitative data were presented as the mean with the standard error of the mean. Cell viability and percentage of activated cells were analysed using Fisher’s exact test. The number of cells adhered and the spreading area of the cells were analysed using a t-test. Statistical comparisons of cytokine release were performed using the one-way analysis of variance (ANOVA) with Tukey’s multiple comparisons test. All statistical analyses were performed with the GraphPad PRISM software (6.01).

## 5. Conclusions

In this study, we demonstrated the biocompatibility of ZnO NGs for smooth muscle cells and skeletal muscle cells. Both cell types were able to adhere, proliferate and differentiate on ZnO NGs. In addition, the ions released over time from the NGs did not affect the viability of muscle cells and did not induce an in vitro inflammatory response. Regarding the piezoelectric stimulation, the electromechanical interaction of NGs and cells was able to stimulate smooth muscle cells by inducing intracellular calcium transients, but no effect was observed on skeletal muscle cells. In single skeletal myotubes, the ZnO NGs simultaneously stimulate different regions of the cell randomly, and the lack of a unidirectional electric field interferes with contraction. The different response of two muscle cell types to the same ZnO NG indicates that the NG distribution and morphology should be custom designed and optimized according to the potential tissue of application. Although the use of electrical stimulation on muscle cells has been extensively studied, the effect of local electric fields at a cell-scale without applying external stimulation has a high upcoming potential. The work herein paves the way for future NG development able to stimulate muscle cells by local electrical impulses.

## Data Availability

Data is contained within the article or Supplementary Material.

## Supplementary Materials

The following are available online at https://www.mdpi.com/article/10.3390/ijms23010432/s1.
