# Supplementary figures and images for: Biocompatibility and Electrical Stimulation of Skeletal and Smooth Muscle Cells Cultured on Piezoelectric Nanogenerators
Source: Int J Mol Sci. 2021 Dec 31;23(1):432. doi: 10.3390/ijms23010432 (PMC8745485; doi:10.3390/ijms23010432)

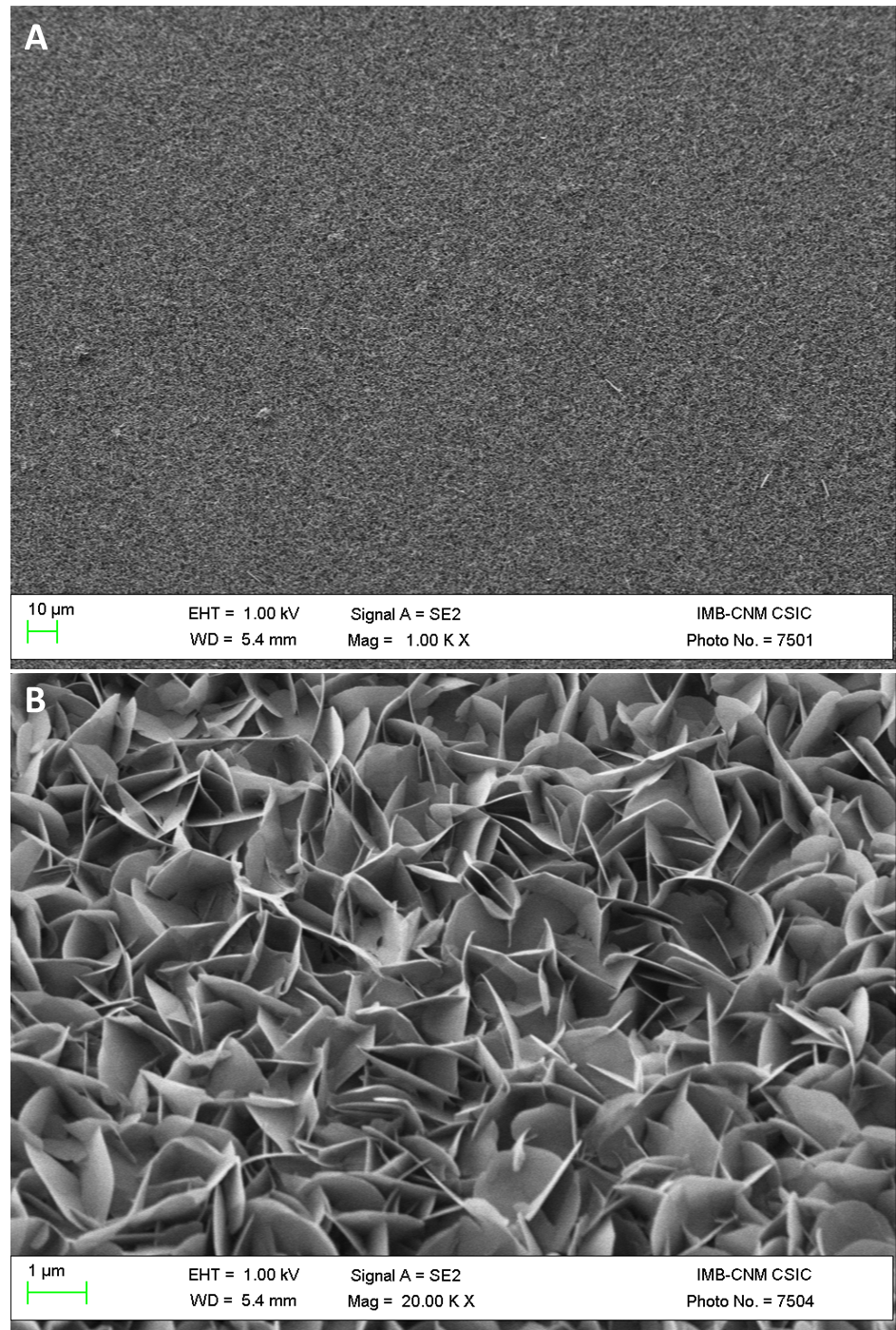

Supplement: Supplementary file 1 [file ijms-23-00432-s001.zip › Productiondata - copia/FIGS1.png]

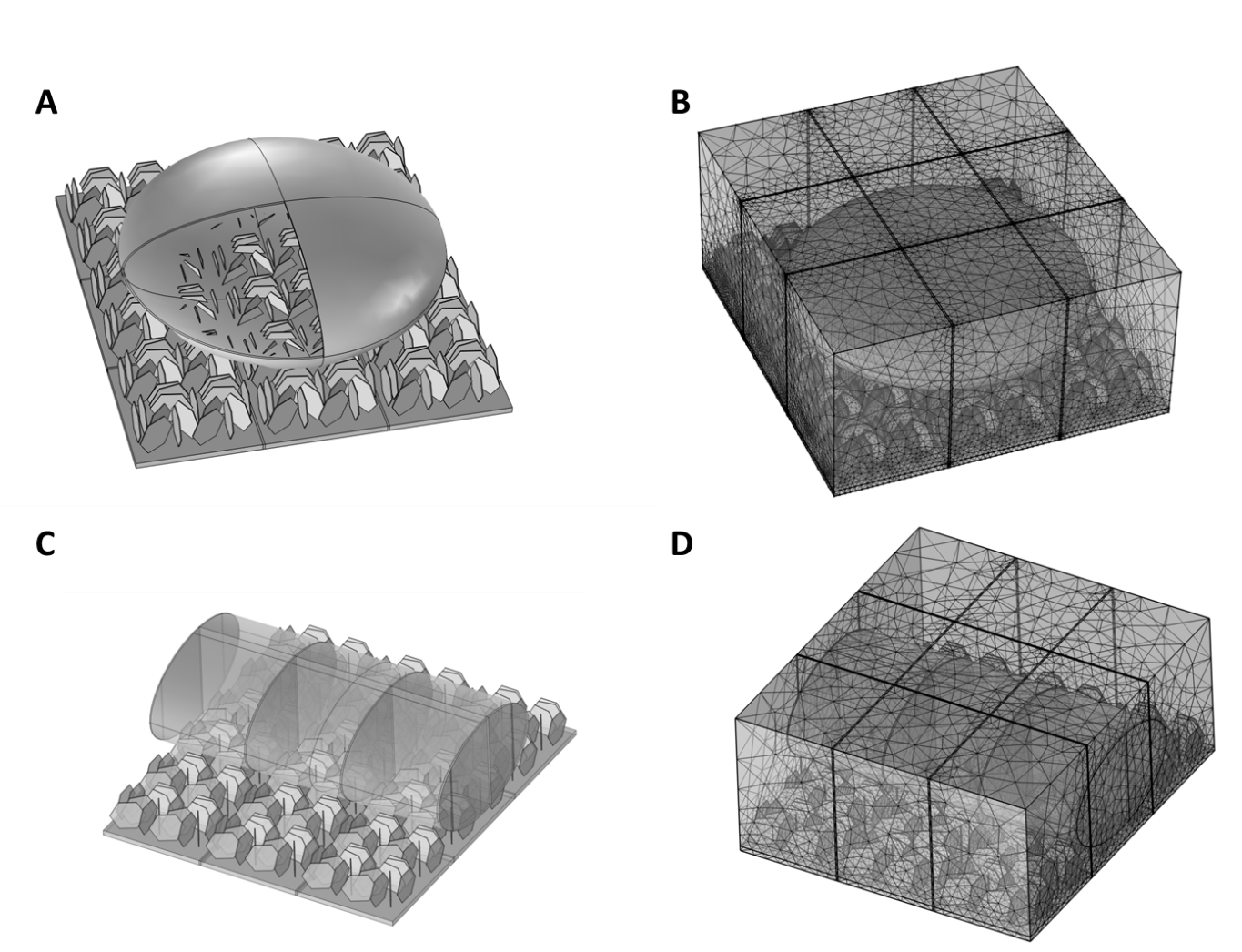

Supplement: Supplementary file 1 [file ijms-23-00432-s001.zip › Productiondata - copia/FIGS2.png]
